# Human papillomavirus 16 mitigates *Sneathia vaginalis*-induced damage to cervical keratinocytes

**DOI:** 10.1128/msphere.00152-25

**Published:** 2025-07-01

**Authors:** Phoebe V. Bridy, Jasmine C. Cruz, Jada L. Covington, Taharah I. Islam, Catherine E. Hadley, Kayla Tran, Rachel Fry, Bradley A. Sheffield, Myrna Serrano, Gregory A. Buck, Jinlei Zhao, Katherine Y. Tossas, Craig Meyers, Iain M. Morgan, Claire D. James, Kimberly K. Jefferson

**Affiliations:** 1 Department of Microbiology and Immunology, Virginia Commonwealth University 542826, Richmond, Virginia, USA; 2 Philips Institute for Oral Health Research, School of Dentistry, Richmond, Virginia, USA; 3 Center for Microbiome Engineering and Data Analysis, Virginia Commonwealth University 6889, Richmond, Virginia, USA; 4 Department of Obstetrics and Gynecology, Virginia Commonwealth University 721690, Richmond, Virginia, USA; 5 Virginia Commonwealth University Massey Comprehensive Cancer Center 172856, Richmond, Virginia, USA; 6 Department of Social and Behavioral Sciences, Virginia Commonwealth University 6889, Richmond, Virginia, USA; 7 Department of Microbiology and Immunology, Pennsylvania State College of Medicine 219261 , Hershey, Pennsylvania, USA; University of Michigan, Ann Arbor, Michigan, USA

**Keywords:** vaginal microbiome, preterm birth, polymicrobial, HPV, *Sneathia*

## Abstract

*Sneathia vaginalis* is a bacterial component of the vaginal microbiome that is of clinical interest because of its association with preterm birth and other obstetric complications. It produces a cytotoxin, but little is known about the mechanism through which it kills epithelial cells or the role that cytotoxicity plays in bacterial survival. Recent microbiome studies demonstrate an association between *S. vaginalis* and human papillomavirus (HPV) within the female reproductive tract, suggesting that HPV and *S. vaginalis* could interact in some way within this shared niche. We analyzed 16S rRNA survey and HPV typing data from our Vaginal Human Microbiome Project and found, in agreement with other reports, that *S. vaginalis* was associated with HPV infection. To test the hypothesis that HPV promotes the growth of *S. vaginalis*, growth and cytotoxicity of *S. vaginalis* in co-culture with HPV16-positive and HPV-negative human cervical keratinocytes (HCK) were quantitatively assessed. Organotypic HCK rafts expressing HPV16 were more resistant to *S. vaginalis*-induced damage, as assessed by histology, and supported increased bacterial growth relative to HPV-negative HCK rafts. When *S. vaginalis* was co-cultured with HPV16-positive and HPV-negative HCK monolayers, cytotoxicity was observed in both HPV16-positive and HPV-negative cells, but HPV16-positive cells were more resistant to the toxic effects of the bacteria and supported bacterial growth for an extended period of time. In conclusion, HPV16 may protect cervical keratinocytes from the cytotoxic effects of *S. vaginalis*, preventing the eradication of colonized cells and supporting bacterial growth, and this could underlie the association between *S. vaginalis* and HPV *in vivo*.

**IMPORTANCE:**

*Sneathia vaginalis* (*S. vaginalis*) is a bacterial species that lives in the human vagina and can cause complications during pregnancy if it invades the uterus. It is capable of killing cervical epithelial cells. Human papillomaviruses (HPV) are sexually transmitted viruses that can cause genital lesions and cervical cancer. Recently, multiple reports describe an association between *S. vaginalis* and HPV. This study used cultured cervical epithelial cells expressing the high-risk HPV type, HPV16, and HPV-negative cells to determine whether HPV promotes the growth of *S. vaginalis*. We found that HPV16 promotes the survival of cervical epithelial cells that are exposed to *S. vaginalis*. Survival of cervical epithelial cells may benefit the growth of *S. vaginalis*, which adhere to and feed off of these cells to survive in the female reproductive tract.

## INTRODUCTION

*Sneathia vaginalis* is a gram-negative anaerobic streptobacillus and a component of the vaginal and anal microbiomes (1, 2). It is highly fastidious, likely due to its reduced genome size, and it requires either human serum or co-culture with a human epithelial monolayer for growth (2). *Sneathia* species are strongly linked to preterm birth, preterm premature rupture of membranes (PPROM), and other obstetric and gynecologic clinical problems (3–9). Multiple studies have confirmed that *S. vaginalis* is one of the most common bacteria to infect the amniotic fluid, particularly in people with PPROM (5, 7, 9). Importantly, we and others have found that *S. vaginalis* is significantly more abundant in the vaginal microbiome of Hispanic and Black people: groups who are also disproportionately affected by preterm birth (10–12). *Sneathia* species have also been reported as the causative agent in cases of neonatal meningitis (13), peripartum bacteremia (14), late miscarriage and post-partum renal abscess (15), tubo-ovarian abscess (16), and salpingitis (17).

While the field of *S. vaginalis* pathogenesis is still in its infancy, a few clues to its influence on the host have emerged. It produces a large pore-forming toxin, Cytopathogenic Toxin A, which lyses human red blood cells and permeabilizes the plasma membrane of human epithelial cells (18). In addition to the direct damage caused by the toxin, this could result in the release of damage-associated molecular patterns that promote stress and inflammation. Co-culture of vaginal bacteria with a three-dimensional organotypic cervix revealed that *S. vaginalis* adheres to host cells and induces a unique inflammatory mediator signature, affects genes involved in epithelial barrier function and stress, and induces accumulation of the oncometabolite, 2-hydroxyglutarate (19). The announcement of the second generation of the nonredundant vaginal microbiome gene catalog (VIRGO2) reported that the expressions of genes related to carbohydrate metabolism that were capable of discriminating between lactobacillus-dominant and non-dominant community state types (CST) were largely from the genus *Sneathia* (20). Together, these results suggest that despite the fact that *Sneathia* rarely dominates the vaginal microbiome, it may play a major role in altering the vaginal environment and damaging host tissues.

Human papillomaviruses (HPVs) are a family of small, nonenveloped DNA viruses. Alpha-papillomaviruses are capable of infecting mucosal epithelial cells within the male and female genital tracts, and HPV is the most common sexually transmitted pathogen (21). Approximately 40 different types of HPV are capable of infecting the reproductive tract, and some of these, the so-called “high-risk” types, are responsible for the majority of cervical carcinomas. Of the high-risk types, HPV16 and 18 are the most important clinically as they cause ~70% of cervical carcinomas (22). Infection with high-risk HPV typically resolves without the development of cervical cancer, but if the virus is able to persist, it can promote progression to cancer. The E6 and E7 proteins are multifunctional, and their oncogenic potential stems, in part, through degradation of the tumor suppressors p53 and pRB (23). In addition to its well-characterized role in cervical cancer, HPV has been shown to have a significant association with preterm birth (24, 25). It is not known whether this association is one of a direct causation or indirect causation through some other variable, such as the microbiome of the female reproductive tract.

Recently, multiple studies have reported that *S. vaginalis* is significantly associated with HPV within the cervicovaginal microbiome (12, 26–31) and in the anal microbiome of HIV +males who develop HR-HPV-mediated anal cancer (32). A twin study found that while the vaginal microbiomes of twins were more similar than those of unrelated individuals, twins who were discordant for HPV infection differed, in that the HPV^+^ twins had higher levels of *S. vaginalis* (30). Multiple studies have found higher levels of *S. vaginalis* associated with squamous intraepithelial lesions (SIL) (27, 29) (33), while another study found it to be associated with both SIL and invasive cervical carcinoma (12). In subjects with cervical intraepithelial neoplasia (CIN), *S. vaginalis* has also been linked to overexpression of HPV genes E6 and E7, the oncogenes encoded in the HPV genome (34). In a prospective study including 103 non-pregnant, premenopausal HPV + women scheduled for CIN resection and 39 healthy controls, vaginal samples for microbiome analysis were taken prior to and 6 months after surgical resection of cervical neoplasia. The study found that the abundance of *S. vaginalis* was significantly higher in CIN participants versus controls, but there was not a significant difference between the treatment group following surgery relative to controls, suggesting cause and effect: that removal of HPV-infected cervical cells reduced the abundance of *S. vaginalis* (26). Multiple recent systematic reviews and meta-analyses consolidating these reports support the consistent significant association between *Sneathia* and HPV, CIN, and cervical carcinoma (35–37).

Certain ethnic groups, including Hispanic/Latina (35), Native American (38), and Black (39) people, are at increased risk for HPV infection and cervical carcinoma relative to White and Asian women. Specific members of the vaginal microbiome, including *Sneathia*, could contribute to this health disparity. For instance, *Sneathia* is enriched in the cervicovaginal microbiomes of Hispanic/Latina people infected with HPV, in those with cervical neoplasia, and in those with cervical carcinoma (12, 35). A study investigated the association between HPV and the vaginal microbiome in Native Americans, and, while the study did not detect any statistically significant associations between specific vaginal taxa and HPV (likely because of small sample size), the vaginal microbiomes of Native Americans were highly enriched for *Sneathia* (38). A study of Black South Africans detected *Sneathia* enrichment using LeFSe analysis in those infected with high-risk HPV (40). Despite lower rates of HPV infection and cervical cancer among White and Asian people, *Sneathia* is also enriched in these populations (12, 41).

It is important to understand the relationship between *Sneathia* and HPV because the damaging substances that *Sneathia* produces, along with the inflammation that it causes, could increase the risk of HPV persistence and of developing cervical carcinoma. Conversely, if HPV promotes the growth of *Sneathia*, then this could increase the risk for preterm birth and other pregnancy complications. No studies to date have included a model system to isolate these two pathogens (*S. vaginalis* and HPV) and examine, under controlled conditions, what the prospective analysis suggests: that HPV affords some benefit to *S. vaginalis*. The goal of the proposed study was to test this hypothesis and characterize the mechanistic relationship between the two pathogens.

## RESULTS

### Association between *S. vaginalis* and high-risk and low-risk HPV

To investigate the association between *S. vaginalis* and HPV, we examined 16S rRNA data and HPV typing data from a total of 2,093 nonpregnant subjects from the Vaginal Human Microbiome Project (VaHMP). Within the cohort, HPV was detected in 756 subjects and not detected in 1,337 subjects. HPV types that have been reported as high-risk types (HR-HPV) were detected in 394 samples, and HPV types that have been reported as low-risk types (LR-HPV) were detected in 477 samples. Of these 477 LR-HPV+ samples, 158 also contained at least one HR-HPV type, and the remaining 319 were negative for HR-HPV. We used the χ test to determine whether there was an association between HPV detection and *S. vaginalis* detection. As shown in Table 1, there was a significant association between the presence of HR-HPV and *S. vaginalis* and a significant association between the presence of LR-HPV and *S. vaginalis*. We stratified the cohort by self-reported race to investigate whether the association between HPV and *S. vaginalis* was affected by this variable. The χ test was used for all groups, except the Asian race, which was analyzed using the Fisher exact test due to the small subject number. When stratified by race, only subjects self-identifying as Black, the race category with the largest sample number, exhibited an association between HPV and *S. vaginalis*.

**TABLE 1:** Relationship between HPV detection and *S. vaginalis*^*a*^

	HPV-ND/ Sv-ND	HPV-ND/ SvD	HPV-D/ Sv-ND	HPV-D/ SvD	*P*-value
All HPV types; all races	*N* = 781	*N* = 556 Median = 202 IQR = 1350	*N* = 369	*N* = 387 Median = 175 IQR = 1439	*X^2^* = 17.61, *P* < **0.000027**
HR-HPV; all races	*N* = 959	*N* = 740 Median = 206 IQR = 1148	*N* = 191	*N* = 203 Median = 149 IQR = 1103	*X^2^* = 7.88, *P* = **0.004989**
LR-HPV; all races	*N* = 919	*N* = 697 Median = 184 IQR = 1367	*N* = 231	*N* = 246 Median = 180 IQR = 1447	*X^2^* = 10.26, *P* = **0.001358**
LR-HPV (HR-HPV-ND); all races	*N* = 803	*N* = 577 Median = 186 IQR = 1350	*N* = 156	*N* = 163 Median = 253 IQR = 1712	*X^2^* = 8.71, *P* = **0.003159**
All HPV types; race = Black	*N* = 331	*N* = 365 Median = 313 IQR = 1743	*N* = 171	*N* = 273 Median = 253 IQR = 1661	*X^2^* = 9.00, *P* = **0.002704**
All HPV types; race = Asian	*N* = 17	*N* = 3	*N* = 3	*N* = 1	Fisher exact test *P* = 0.544
All HPV types; race = Hispanic	*N* = 92	*N* = 30 Median = 132 IQR = 2348	*N* = 40	*N* = 22 Median = 54 IQR = 4476	*X^2^* = 1.90, *P* = 0.12085
All HPV types; race = White	*N* = 261	*N* = 97 Median = 44 IQR = 302	*N* = 118	*N* = 60 Median = 47 IQR = 216	*X^2^* = 2.20, *P* = 0.137914
All HPV types; other	*N* = 80	*N* = 61 Median = 172 IQR = 1891	*N* = 37	*N* = 31 Median = 60 IQR = 332	*X^2^* = 0.1007, *P* = 0.75984

^*a*^ χ analysis with Yates correction was used to investigate the association between HPV presence and *S. vaginalis* presence. HPV-ND, HPV reads ≤ 55. HPV-D, HPV reads >55. Sv-ND, Sv reads ≤ 9. Sv-D, Sv reads >9. Median, median number of Sv reads. IQR, interquartile range. *P* values shown in bold are considered statistically significant.

### *S. vaginalis* destroys the HPV-negative organotypic cervical keratinocyte epithelium, but the HPV16+ epithelium is resistant to damage

**Fig 1:**
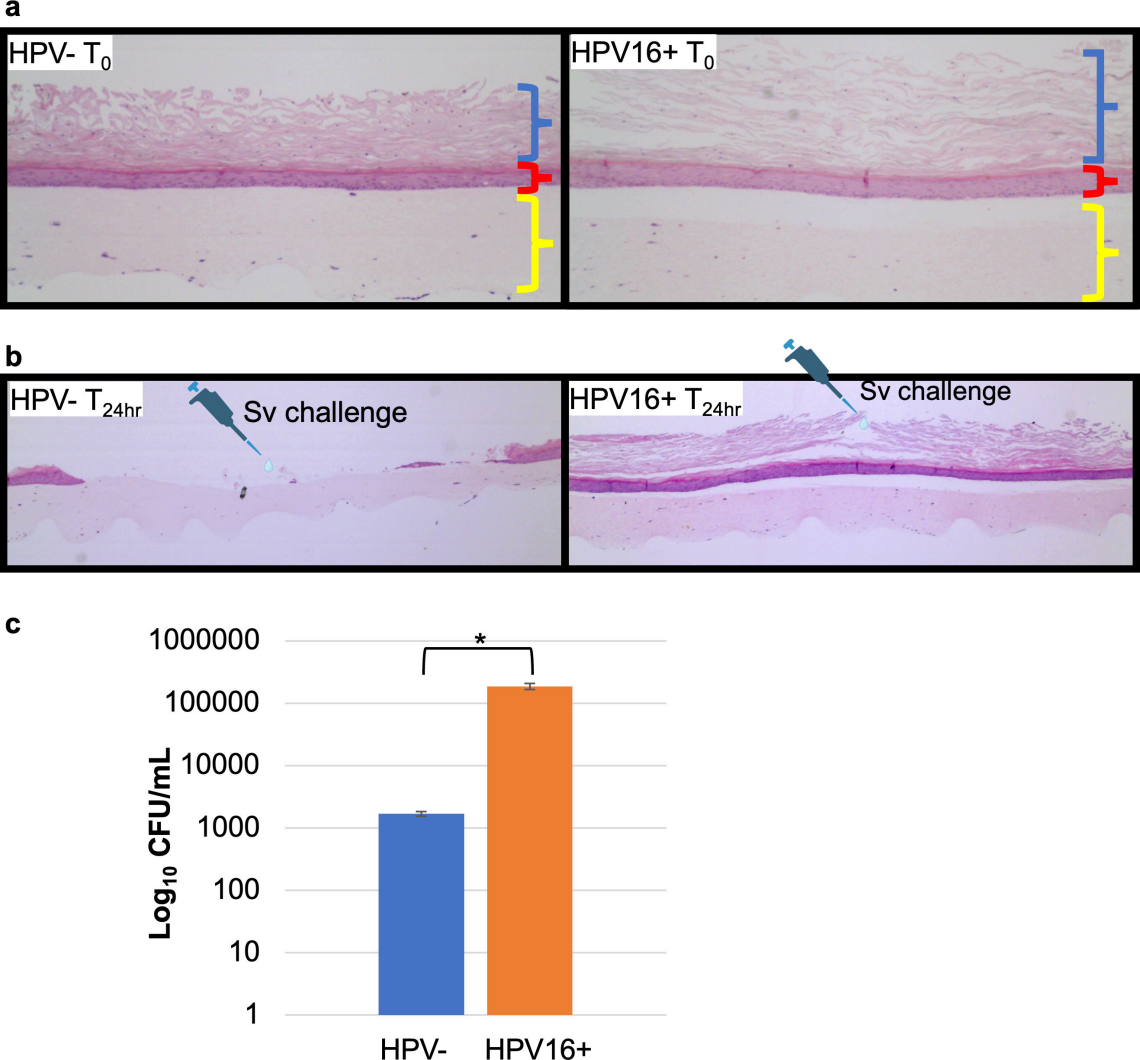
HPV16 mitigates damage to organotypic cervical epithelial cultures and results in increased bacterial growth. (a) Untreated HPV- and HPV16+ HCK rafts. The HPV- HCK formed an epithelium ~15 layers thick, with ~5 layers in the superficial layer (blue bracket) and ~10 layers in the mid-zone and parabasal layers (red bracket). The yellow bracket denotes the collagen plug. HPV16+ HCK formed an epithelium with ~26 layers, of which ~ 12 were in the superficial zone and ~14 in the mid-zone and parabasal layers. (b) *S. vaginalis* was inoculated in the center of the raft, and the rafts were incubated for 24 hours. The epithelium detached in the region proximal to the inoculation site in HPV- rafts, while only modest damage to superficial epithelia in HPV16+ rafts was observed. (c) Rafts were homogenized and plated to enumerate viable bacteria. Approximately 100-fold more colony-forming units were detected in HPV16+ raft cultures relative to HPV- (Student’s *t*-test *P* value < 0.0001).

Because previous studies suggest an association between HPV and *S. vaginalis*, a finding supported in this study by the VaHMP data, we investigated the effect of HPV on *S. vaginalis* colonization *in vitro*. Three-dimensional organotypic raft tissues composed of HPV-negative (HPV-) and HPV-positive (HPV16+) primary HCK were cultured. Raft tissues were inoculated with ~10^6^ live *S. vaginalis* by spotting the bacteria carefully into the center of the tissue. As *S. vaginalis* is nonmotile, the infection would be expected to remain somewhat localized, allowing us to observe the effects of the bacteria on the tissue proximal to the spotted area and compare it to the tissue distal to the inoculation site. After 24 hours, collagen plugs and overlaying epithelia were rinsed in PBS, formalin-fixed and paraffin-embedded (FFPE), and 10 µm sections were stained with hematoxylin and eosin. The full thickness of the HPV- epithelia disappeared, either through detachment from the collagen plug or through disintegration of the cells, in the region of the inoculation site. In contrast, the inoculation site of the HPV16+ tissue sustained only minor visible damage within the superficial layer, while the parabasal layer remained intact and adherent to the collagen plug (Fig. 1a and b). Separate raft culture samples were homogenized and plated to enumerate viable bacteria. As shown in Fig. 1c, HPV16+ rafts contained ~100-fold more viable bacteria relative to HPV- rafts (Student's *t*-test *P* < 0.0001).

### *S. vaginalis* causes disintegration rather than detachment of HPV-HCK monolayers

**Fig 2:**
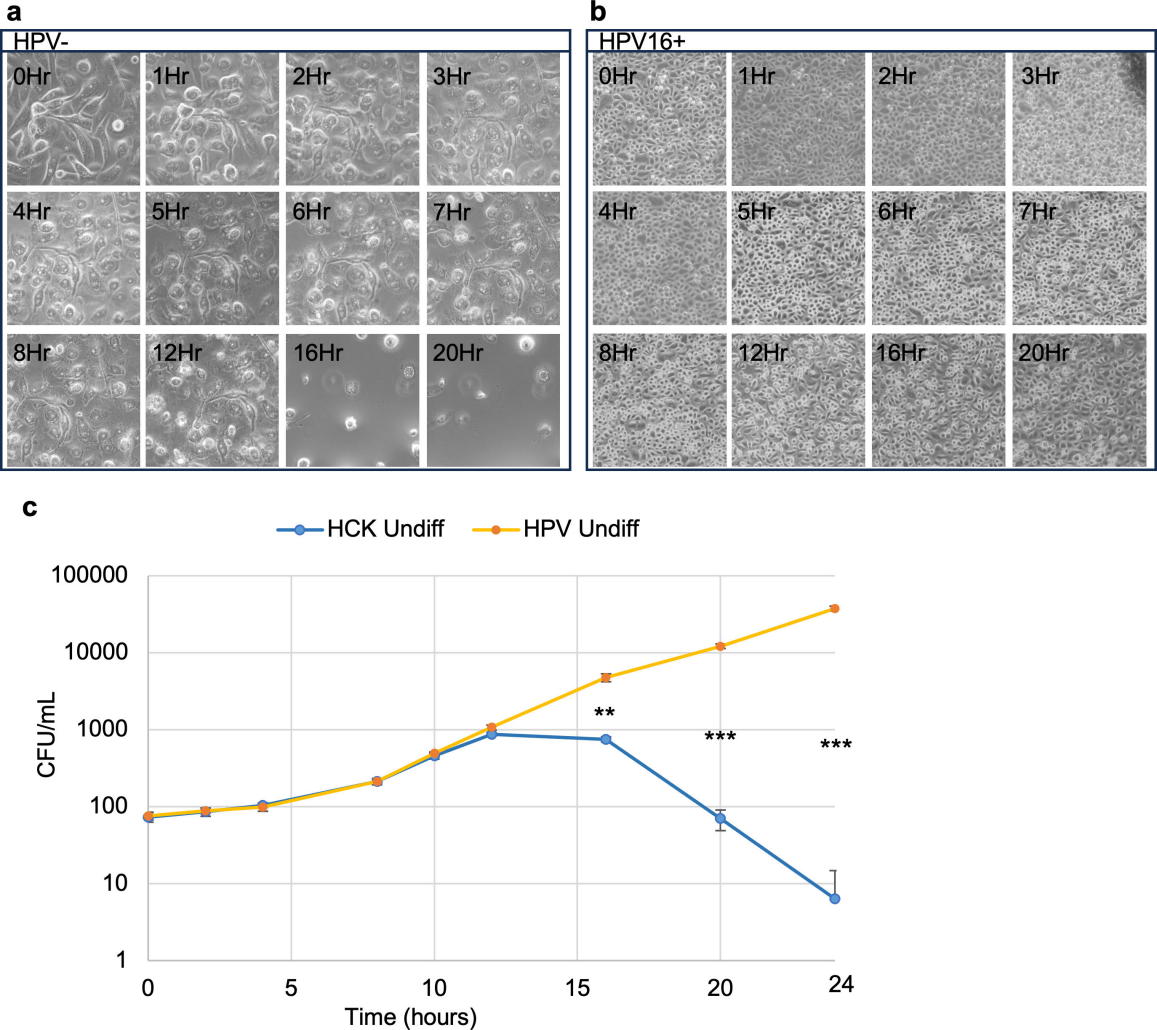
HPV16+ HCK monolayers are more resistant to *S. vaginalis*-mediated cytotoxicity. (**a, b, and c**) HPV- and HPV16+ HCK monolayers were challenged with a *S. vaginalis* MOI = 100. (**a and b**) The same region of the culture vessel was imaged by light microscopy over time to assess morphologic changes. (**c**) Adherent *S. vaginalis* were enumerated over time by counting colony-forming units. Markers = average and error bars = SD of three technical replicates. Assays were repeated three times. Significance was detected using an unpaired *t* test: * *P* ≤ 0.01, ** *P* ≤ 0.001, and *** *P* ≤ 0.0001.

We reasoned that loss of the organotypic HCK epithelium in response to *S. vaginalis* could be a result of detachment of the cells from the matrix, similar to the way that superficial epithelial cells desquamate *in vivo* or to disintegration of individual cells, through necrosis or programmed cell death. To more carefully examine the effect of *S. vaginalis* on HCK monolayers, we monitored co-cultures over time by light microscopy. Morphologic changes in HPV- HCK were observed within an hour (Fig. 2a). In contrast, no morphological changes in HPV16+ HCK were detected by light microscopy until the 5 hour time point (Fig. 2b). Rounded and degraded HPV- cells did not detach from the vessel surface, but after 16 hours, most of the cells had completely disintegrated (Fig. 2a). This suggests that *S. vaginalis* does not cause detachment but rather epithelial cell degradation and supports the results from raft co-culture experiments, in that HPV+ HCK were resistant to its damaging effects. Adherent, viable bacteria in co-culture with HCK monolayers were enumerated over the time course as well. At the 16 hour time point, after destruction of the HPV- monolayers had occurred, the number of adherent bacteria declined rapidly, whereas viable bacterial counts continued to increase in co-culture with HPV16+ HCK monolayers (Fig. 2c).

### HPV16+ HCK monolayers are more resistant to *S. vaginalis*-mediated cytotoxicity

**Fig 3:**
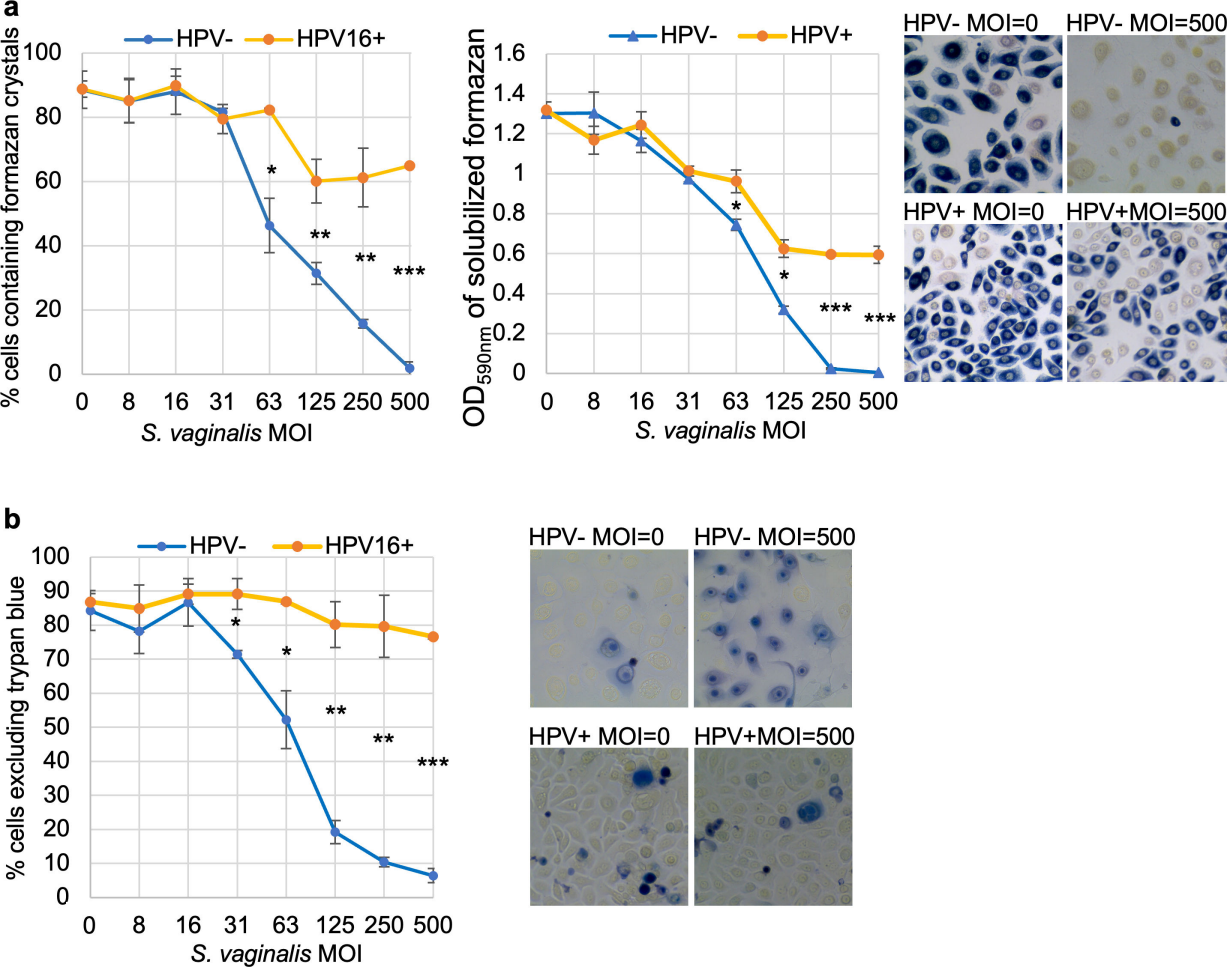
HPV16+ HCK are more resistant to *S. vaginalis*-mediated cytotoxicity. (a) MTT assays were used to assess metabolic activity in HCK challenged with *S. vaginalis* for 2 hours. The percent of cells that were metabolically active (contained formazan crystals) relative to total cells, total formazan crystal formation (OD_590nm_ of DMSO-solubilized formazan), and phase contrast microscopic images showing formazan crystal accumulation in untreated HCK and cells treated with an MOI = 500 *S*. *vaginalis* are shown. (b) Trypan blue exclusion was used to assess membrane integrity in the HCK. The percent of cells permeable to trypan blue relative to total cells and brightfield microscopic images showing trypan blue staining in untreated HCK and cells treated with an MOI = 500 *S*. *vaginalis* are shown. Markers = average and error bars = SD of three technical replicates. Assays were repeated three times. Significance was detected using the unpaired *t* test: * *P* ≤ 0.01, ** *P* ≤ 0.001, and *** *P* ≤ 0.0001.

To quantify *S. vaginalis*-induced cytotoxicity in response to different doses of *S. vaginalis* and to learn more about the nature of the damage induced by the bacteria, we quantified NADPH-dependent oxidoreductase activity (MTT assay) and membrane integrity by trypan blue exclusion. At MOI = 62.5, 125, 250, and 500, statistically significantly higher percentages of HPV16+ HCK produced formazan crystals relative to HPV- HCK, and significantly higher total formazan formation was detected spectrophotometrically in HPV16+ HCK relative to HPV- HCK (Fig. 3a). Similarly, at MOI = 62.5, 125, 250, and 500, statistically significantly lower percentages of HPV16+ HCK were permeabilized to trypan blue (Fig. 3b).

## DISCUSSION

Recent microbiome studies have revealed an association between certain vaginal bacterial taxa and HPV (12, 30, 31, 41–45). We found, upon examining a cohort of 2,096 participants from the Human Vaginal Microbiome Project (VaHMP), that, in agreement with other studies, there is a significant association between HPV and the presence of *S. vaginalis*. In our study, however, there was no association between the number of *S. vaginalis* reads and presence/absence of HPV and no correlation between *S. vaginalis* read number and HPV read number. It is possible that HPV promotes *S. vaginalis* colonization or survival in the female reproductive tract, but that other factors, such as the relative abundances of other microbial species, play a greater role in the absolute numbers of *S. vaginalis*. More specifically, vaginal lactobacilli are consistently found to have a strong negative correlation with *Sneathia* species (46–48) and dominate the healthy microbiome, even in people infected with HPV. It is also possible that there is a poor correlation between the absolute numbers of *S. vaginalis* and the read number as the samples collected for this study were not standardized for the volume of the vaginal fluid collected.

We examined the association between HPV and *S. vaginalis* within different racial groups. However, when the data were stratified by race, an association was only detected within study subjects who self-reported as Black. As the largest number of subjects were in this race category (*n* = 1,140), the failure to detect an association between HPV and *S. vaginalis* in the other groups may have been due to insufficient statistical power from the lower numbers. The study cohort contained 184 Hispanic subjects, 536 White subjects, and only 24 Asian participants. In support of the study being underpowered, other studies have detected an association between HPV and *Sneathia* in these populations (12, 35, 40)

Very few interactions between viral and bacterial pathogens are understood at a mechanistic level. We hypothesized that the positive association between HPV and *S. vaginalis* was imparted by a benefit of the viral infection of cervical keratinocytes to the fitness of *S. vaginalis*. To begin to address this, we used 3-D epithelial models. The HPV life cycle is intricately linked to epithelial differentiation. Viral particles infect the basal layer of the epithelium, whereupon viral transcription and replication occur. Viral oncogenes E6 and E7 combine to promote host cell proliferation and differentiation. Ultimately, completed viral particles egress from the fully differentiated upper layers of the epithelium. Throughout the viral life cycle, the viral genome is maintained as an extrachromosomal ~8 kbp plasmid. This process is mimicked *in vitro* by organotypic rafting in which HPV-immortalized keratinocytes are seeded onto a collagen plug infused with fibroblasts (a stroma equivalent). Following the lifting of the epithelial cells to the liquid-air interface (day 7), differentiation of the HCK occurs in 14 days, producing an epithelium equivalent (49). We found that rafts composed of HPV16+ HCK were resistant to *S. vaginalis*-mediated damage and that they supported the presence of a much larger number of associated viable bacteria. In support of these findings, the Herbst-Kralovetz group reported previously that three-dimensional organotypic cultures of cervical keratinocytes immortalized with the HPV E6 and E7 genes were capable of supporting the growth of *S. vaginalis* (19, 50).

We next investigated the effect of HPV16 on *S. vaginalis*-induced cytotoxicity using HCK monolayers. HPV16 protected cervical keratinocytes from the morphologic changes seen in HPV- HCK, and as the HPV- HCK monolayer was destroyed, the number of adherent *S. vaginalis* declined as well, either because all of the bacteria adhered to the cells and detached with them or because the dead epithelial cells were no longer capable of supporting bacterial growth. Cytotoxicity in *S. vaginalis* is mediated by the CptA toxin (18, 51), but the moderation of cytotoxicity by HPV has not been previously reported. *S. vaginalis* is devoid of genes encoding the synthesis of many basic building blocks including most amino acids, nucleotides, and lipids, but is equipped with salvage pathways, making it fastidious and reliant on the host (2). The fastidious nature of *S. vaginalis* may necessitate that the bacteria grow on or inside of host cells. Thus, host cell death could serve as a host defense by eradicating infected cells from the vagina, and conversely, suppression of cell death by HPV would be expected to increase the abundance levels of the bacteria, a phenomenon that bears out in microbiome analyses.

A limitation of this study is that it did not investigate the effect of *S. vaginalis* challenge on HPV integration or gene expression. *S. vaginalis*-induced stress could affect HPV persistence and/or oncogenesis, and this hypothesis is supported both by the microbiome studies and by the controlled *in vitro* study from the Herbst-Kralovetz group in which *S. vaginalis* promoted stress and oncometabolite accumulation in 3-D cervical cultures (19). Our study examined the effect of HPV on *S. vaginalis,* but the reciprocal effects of *S. vaginalis* on HPV replication, persistence, and oncogenesis should be addressed in a subsequent study. A better understanding of the mechanism of cell death that *S. vaginalis* induces in keratinocytes and how HPV might abrogate that process is also an important next step in understanding the relationship between these two pathogens.

## MATERIALS AND METHODS

### Participant enrollment, informed consent, and sample collection

Subjects for this study included 2,093 nonpregnant participants enrolled in the Vaginal Human Microbiome Project at VCU (VaHMP). Participants for VaHMP were recruited from outpatient clinics at the Virginia Commonwealth University Medical Center and the Virginia Department of Health following written, informed consent from 2009 to 2013 (52). Participants were informed that they could withdraw from the study at any time and that providing samples was at their sole discretion. Participants filled out a detailed questionnaire and self-reported race and other demographic characteristics. Inclusion criteria for VaHMP included female sex, at least 18 years of age, and the ability and willingness to provide informed consent and undergo a vaginal examination using a speculum. The inclusion criterion for the subset included in this study included not being pregnant.

### Sample collection and processing

Clinicians used CultureSwab EZ polyurethane foam swabs (BD) to obtain specimens from the mid-vaginal wall during a speculum examination. DNA was extracted from the swabs within 4  h of collection using a Powersoil kit (MoBio). Surveys of the 16S rRNA genes present in the samples were generated as part of the Vaginal Human Microbiome Project (53).

### 16S rRNA gene survey

The V1–V3 hypervariable regions of the bacterial 16S rRNA gene were amplified by PCR using barcoded primers, as previously reported (10). PCR products were sequenced using the Roche 454 GS FLX Titanium platform. These data were generated as part of the Vaginal Human Microbiome Project (53). Raw sequence data from the project are available from the Short Read Archive at NCBI (projectID phs000256) (53). We used a deep sequencing approach with a median of 24,030 reads/sample. Samples with fewer than 1,000 reads were excluded from the analysis.

Reads that met the following criteria were processed: (i) valid primer and multiplex identifier sequences were observed; (ii) less than 10% of base calls had a quality score less than 10; (iii) the average quality score was greater than Q20; and iv) the read length was between 200 and 540 bases. Sequences were classified using STIRRUPS, an analysis platform that employs the USEARCH algorithm combined with a curated vaginal 16S rRNA gene database (52, 54).

### PCR typing using the HPV L1 gene fragment

The HPV status and type(s) of the samples were determined in a two-step polymerase chain reaction (PCR). HPV DNA was amplified using consensus primer sets to target a 452 bp region of the L1 gene (corresponding to flanking nucleotide positions 946/1397 on the HPV16 L1 gene).

The first PCR was performed to amplify the target region using L1-specific primers with partial Illumina adapter sequences (shown in italics). The forward primer was HPV-01F (5′ *TCG TCG GCA GCG TCA GAT GTG TAT AAG AGA CAG* GCA CAG GGM CAH AAY AAT GG 3′) and reverse primer HPV-6R (5′ *GTC TCG TGG GCT CGG AGA TGT GTA TAA GAG ACA G*NC GTC CCA AAG GRW AYT GAT C 3′). Each reaction was carried out in a total volume of 26 µL containing approximately 20 ng of the DNA template, 0.2 µM of forward and reverse primers, 10 mM dNTPs, 3.75 U of Thermo Fisher AmpliTaq DNA polymerase, 1.5 µL of 25 mM MgCl_2_ and 2.5 µL of the 10X supplied buffer. The PCR cycling conditions were performed as described previously (55): initial denaturation at 95°C for 9 min, followed by 40 cycles of denaturation at 95°C for 60 s, annealing at 55°C for 60 s, and extension at 72°C for 60 s, with a final extension at 72°C for 5 min. Amplified DNA was cleaned up using 0.8× Agencourt AMPure XP (Beckman Coulter Genomics) following the standard manufacturer’s protocol and resuspended in 25 µL of Teknova DNA Suspension buffer. A second PCR was performed to attach sample-specific barcodes and full Illumina sequencing adapters. This reaction used 5 µL of the purified first-step PCR product diluted 1:10 as a template, combined with 0.05 µM of Nextera indexing primers, 12.5 µL of New England Biolabs Q5 High-Fidelity 2× Master Mix in a total volume of 25 µL. The thermocycling conditions included an initial denaturation at 98°C for 5 min, followed by 12 cycles of 95°C for 30 s, 62°C for 60 s, and 72°C for 60 s, with a final extension at 72°C for 10 min. Post-PCR amplicons were purified using 0.8× Beckman Coulter AMPure XP beads following the manufacturer’s protocol. DNA concentration was quantified using the Promega QuantiFluor ds DNA System on a Biotek Synergy HT Reader, and the fragment size distribution was assessed using an Agilent TapeStation. Equimolar amounts of indexed amplicons were pooled and submitted for sequencing on an Illumina MiSeq platform using a 600 bp paired-end read configuration.

### Data processing and HPV-type assignment

Raw sequence reads were demultiplexed using sample-specific barcodes, adapter sequences were trimmed, and human reads filtered out using in-house Python scripts. The raw overlapping paired-end sequence data were merged and quality-filtered using MeFiT with a meep (maximum expected error rate) cutoff of 1.0 (56). HPV typing classification was conducted using a custom reference database. High-quality taxonomic assignments at 96% similarity were performed using STIRRUPS (52), employing the USEARCH (57) algorithm combined with the L1 gene database. The reference database consists of 222 officially established HPV genotype sequences downloaded from Karolinska Institute in October 2021. Of the 1,168 samples remaining, 530 were considered HPV +if at least 55 reads were assigned to any HPV type. This value was defined based on the mean number of reads in the negative control samples assigned to any HPV type +2 standard deviations. HPV types 16, 18, 26, 31, 33, 35, 39, 45, 51, 52, 53, 56, 58, 59, 66, 68, 73, and 82 were categorized as high risk. HPV types 6, 11, 32, 40, 42, 44, 54, 57, 61, 62, 70, 71, 72, 74, 81, 83, 84, 86, 87, 89, 90, 102, and 114 were categorized as low risk. HPV types 30, 34, 38, 67, 85, 107, 118, 120, and 124 were categorized as unknown risk.

### Cell lines and culture

Primary human cervical keratinocytes (HCK) were a gift from Dr. Craig Meyers. HCK were cultured in Dermalife-K complete media (Lifeline Technology). The cells were immortalized with HPV16, as described previously (58). Briefly, HPV16 genomes were removed from their parental plasmid using Sph1, re-circularized using T4 ligase (NEB), and transfected into early passage HCK alongside a G418 resistance plasmid, pcDNA3.1. Cells then underwent selection in 100 µg/mL G418 (Sigma-Aldrich) for 14 days and were cultured on a layer of 3T3-J2 fibroblast feeders (Kerafast), which had been pretreated with 4 µg/mL mitomycin C (Roche). Throughout the immortalization process, HCK were cultured in Dermalife-K complete (DLK). They were seeded in plates for experimental analysis. HPV-uninfected HCK were grown in DLK without J2 feeders. Cells were grown in 96-well plates until they reached confluence (approximately 5 × 10^4^ cells per well).

### Organotypic raft culture

Keratinocytes were differentiated via organotypic raft culture, as described previously (58). Briefly, one million cells were seeded onto collagen matrices containing one million J2-3T3 fibroblast feeder cells. Cells were grown to confluency atop the collagen matrices and were then lifted onto wire grids and cultured at the air-liquid interface for 13 days, with media replacement on alternate days. Rafted samples were challenged with ~10^6^ mid-log phase *S. vaginalis* in 5 µL DLK by spotting the inoculum in the center of the rafts, and the co-cultures were incubated at 37°C 5% CO_2_ for 24 hours. After 24 hours, the rafts were rinsed gently in PBS, fixed with formaldehyde (4% v/v), and embedded in paraffin blocks. Four-micrometer sections were cut from each sample and stained with hematoxylin and eosin. Tissue architecture was observed by light microscopy.

### Bacterial culture

*S. vaginalis* strain Sn35 was cultured anaerobically in porcine brain heart infusion (BHI) broth containing 5% fetal bovine serum. To challenge HCK monolayers for time-course assays, ~5 × 10^7^ bacteria in the logarithmic phase were collected by centrifugation at 4,000 × *g* for 4 minutes at 21°C. The pellet was washed once in DLK, collected again by centrifugation, and resuspended in 1 mL fresh DLK, and 100 µL was added to HCK monolayers (~5 × 10^4^ per well). The medium was removed from wells at different time points. The monolayers were rinsed gently one time with 200 µL HBSS and covered in 200 µL fresh HBSS prior to visualization by light microscopy using a 20X objective. To challenge HCK monolayers for cell death analysis, 2 × 10^7^ log-phase bacteria were added to HCK monolayers (~4 × 10^4^ per well) in the first row of 96-well plates to equal an MOI = 500. Twofold dilutions were prepared by transferring 100 µL to each subsequent row, and no bacteria were added to the final row.

### Cell death analysis

To assess the oxidoreductase activity, 15 mg MTT was added to 1.5 mL PBS, covered with foil, and mixed by end-over-end rocking for 2 hours. The MTT solution was filtered through a 0.22 micron filter and diluted with two volumes (3 mL) DLK. After incubating cells with bacteria for 2 hours, the medium was removed, and the monolayers were washed once with PBS, and 100 µL of the media/MTT solution was added per well. After 3 hours in the CO_2_ incubator, the media/MTT solution was removed, and replaced with 100 µL Hanks balanced salt solution (HBSS), and cells were imaged by light microscopy at 20×. The percentage of cells with formazan crystals was calculated as 100× the number of cells containing formazan divided by the total number of cells in one objective view. Formazan crystals were solubilized in 75 µL DMSO, and total formazan was measured as the optical density at 590 nm. For trypan blue staining, the monolayers were treated with bacteria for 4 hours. Following bacterial challenge, the medium was removed, and 100 µL 0.1% trypan blue in phosphate-buffered saline was added to the wells for 1 minute, removed, and the cells were visualized by brightfield light microscopy. Digital images of three separate fields were saved, and the ratio of the unstained to stained cells was counted in each of the three fields and averaged.

## Data Availability

Raw sequence data from the project are available from the Sequence Read Archive at NCBI (project ID phs000256).

## References

[1] Raimondi S, Candeliere F, Amaretti A, Foschi C, Morselli S, Gaspari V, Rossi M, Marangoni A. 2021. Vaginal and anal microbiome during Chlamydia trachomatis infections. Pathogens 10:1347. doi:10.3390/pathogens10101347 34684295 PMC8539191

[2] Harwich MD Jr, Serrano MG, Fettweis JM, Alves JMP, Reimers MA, Buck GA, Jefferson KK, Vaginal Microbiome Consortium (additional members). 2012. Genomic sequence analysis and characterization of Sneathia amnii sp. nov. BMC Genomics 13 Suppl 8:S4. doi:10.1186/1471-2164-13-S8-S4 PMC3535699 23281612

[3] Shukla SK, Meier PR, Mitchell PD, Frank DN, Reed KD. 2002. Leptotrichia amnionii sp. nov., a novel bacterium isolated from the amniotic fluid of a woman after intrauterine fetal demise. J Clin Microbiol 40:3346–3349. doi:10.1128/JCM.40.9.3346-3349.2002 12202577 PMC130742

[4] Gardella C, Riley DE, Hitti J, Agnew K, Krieger JN, Eschenbach D. 2004. Identification and sequencing of bacterial rDNAs in culture-negative amniotic fluid from women in premature labor. Am J Perinatol 21:319–323. doi:10.1055/s-2004-831884 15311367

[5] DiGiulio DB, Romero R, Amogan HP, Kusanovic JP, Bik EM, Gotsch F, Kim CJ, Erez O, Edwin S, Relman DA. 2008. Microbial prevalence, diversity and abundance in amniotic fluid during preterm labor: a molecular and culture-based investigation. PLoS One 3:e3056. doi:10.1371/journal.pone.0003056 18725970 PMC2516597

[6] Han YW, Shen T, Chung P, Buhimschi IA, Buhimschi CS. 2009. Uncultivated bacteria as etiologic agents of intra-amniotic inflammation leading to preterm birth. J Clin Microbiol 47:38–47. doi:10.1128/JCM.01206-08 18971361 PMC2620857

[7] DiGiulio DB, Gervasi M, Romero R, Mazaki-Tovi S, Vaisbuch E, Kusanovic JP, Seok KS, Gómez R, Mittal P, Gotsch F, Chaiworapongsa T, Oyarzún E, Kim CJ, Relman DA. 2010. Microbial invasion of the amniotic cavity in preeclampsia as assessed by cultivation and sequence-based methods. J Perinat Med 38:503–513. doi:10.1515/jpm.2010.078 20482470 PMC3325506

[8] DiGiulio DB. 2012. Diversity of microbes in amniotic fluid. Semin Fetal Neonatal Med 17:2–11. doi:10.1016/j.siny.2011.10.001 22137615

[9] Romero R, Miranda J, Chaemsaithong P, Chaiworapongsa T, Kusanovic JP, Dong Z, Ahmed AI, Shaman M, Lannaman K, Yoon BH, Hassan SS, Kim CJ, Korzeniewski SJ, Yeo L, Kim YM. 2015. Sterile and microbial-associated intra-amniotic inflammation in preterm prelabor rupture of membranes. J Matern Fetal Neonatal Med 28:1394–1409. doi:10.3109/14767058.2014.958463 25190175 PMC5371030

[10] Fettweis JM, Brooks JP, Serrano MG, Sheth NU, Girerd PH, Edwards DJ, Strauss JF, Jefferson KK, Buck GA, the Vaginal Microbiome Consortium. 2014. Differences in vaginal microbiome in African American women versus women of European ancestry. Microbiology (Reading, Engl) 160:2272–2282. doi:10.1099/mic.0.081034-0 PMC4178329 25073854

[11] Serrano MG, Parikh HI, Brooks JP, Edwards DJ, Arodz TJ, Edupuganti L, Huang B, Girerd PH, Bokhari YA, Bradley SP, et al.. 2019. Racioethnic diversity in the dynamics of the vaginal microbiome during pregnancy. Nat Med 25:1001–1011. doi:10.1038/s41591-019-0465-8 31142850 PMC6746180

[12] Łaniewski P, Barnes D, Goulder A, Cui H, Roe DJ, Chase DM, Herbst-Kralovetz MM. 2018. Linking cervicovaginal immune signatures, HPV and microbiota composition in cervical carcinogenesis in non-Hispanic and Hispanic women. Sci Rep 8:7593. doi:10.1038/s41598-018-25879-7 29765068 PMC5954126

[13] Devi U, Bora R, Das JK, Malik V, Mahanta J. 2014. Sneathia species in a case of neonatal meningitis from Northeast India. Oxf Med Case Reports 2014:112–114. doi:10.1093/omcr/omu044 25988049 PMC4369986

[14] De Martino SJ, Mahoudeau I, Brettes JP, Piemont Y, Monteil H, Jaulhac B. 2004. Peripartum bacteremias due to Leptotrichia amnionii and Sneathia sanguinegens, rare causes of fever during and after delivery. J Clin Microbiol 42:5940–5943. doi:10.1128/JCM.42.12.5940-5943.2004 15583348 PMC535221

[15] Thilesen CM, Nicolaidis M, Lökebö JE, Falsen E, Jorde AT, Müller F. 2007. Leptotrichia amnionii, an emerging pathogen of the female urogenital tract. J Clin Microbiol 45:2344–2347. doi:10.1128/JCM.00167-07 17522272 PMC1933011

[16] Gundi VAKB, Desbriere R, La Scola B. 2004. Leptotrichia amnionii and the female reproductive tract. Emerg Infect Dis 10:2056–2057. doi:10.3201/eid1011.031019 16010750 PMC3328984

[17] Hebb JK, Cohen CR, Astete SG, Bukusi EA, Totten PA. 2004. Detection of novel organisms associated with salpingitis, by use of 16S rDNA polymerase chain reaction. J Infect Dis 190:2109–2120. doi:10.1086/425929 15551209

[18] Gentile GL, Rupert AS, Carrasco LI, Garcia EM, Kumar NG, Walsh SW, Jefferson KK. 2020. Identification of a cytopathogenic toxin from Sneathia amnii. J Bacteriol 202:e00162-20. doi:10.1128/JB.00162-20 32291280 PMC7283592

[19] Łaniewski P, Herbst-Kralovetz MM. 2021. Bacterial vaginosis and health-associated bacteria modulate the immunometabolic landscape in 3D model of human cervix. NPJ Biofilms Microbiomes 7:88. doi:10.1038/s41522-021-00259-8 34903740 PMC8669023

[20] France MT, Chaudry I, Rutt L, Quain M, Shirtliff B, McComb E, Maros A, Alizadeh M, Hussain FA, Elovitz MA, Relman DA, Rahman A, Brotman RM, Price J, Kassaro M, Holm JB, Ma B, Ravel J. 2025. VIRGO2: unveiling the functional and ecological complexity of the vaginal microbiome with an enhanced non-redundant gene catalog. bioRxiv:2025.03.04.641479. doi:10.1101/2025.03.04.641479 PMC12800114 41353361

[21] de Villiers E-M, Fauquet C, Broker TR, Bernard H-U, zur Hausen H. 2004. Classification of papillomaviruses. Virology (Auckl) 324:17–27. doi:10.1016/j.virol.2004.03.033 15183049

[22] Wei F, Georges D, Man I, Baussano I, Clifford GM. 2024. Causal attribution of human papillomavirus genotypes to invasive cervical cancer worldwide: a systematic analysis of the global literature. The Lancet 404:435–444. doi:10.1016/S0140-6736(24)01097-3 39097395

[23] Spurgeon ME, Lambert PF. 2020. Mus musculus papillomavirus 1: a new frontier in animal models of papillomavirus pathogenesis. J Virol 94:e00002-20. doi:10.1128/JVI.00002-20 32051276 PMC7163119

[24] Niyibizi J, Mayrand M-H, Audibert F, Monnier P, Brassard P, Laporte L, Lacaille J, Zahreddine M, Bédard M-J, Girard I, Francoeur D, Carceller AM, Lacroix J, Fraser W, Coutlée F, Trottier H, HERITAGE Study Group. 2021. Association between human papillomavirus infection among pregnant women and preterm birth. JAMA Netw Open 4:e2125308. doi:10.1001/jamanetworkopen.2021.25308 34524433 PMC8444026

[25] Kovács D, Szabó A, Hegyi P, Ács N, Keszthelyi M, Sára L, Csirzó Á, Mátrai P, Munnoch K, Nagy R, Bánhidy F. 2024. Association between human papillomavirus and preterm delivery: a systematic review and meta-analysis. Acta Obstet Gynecol Scand 103:1933–1942. doi:10.1111/aogs.14913 39016354 PMC11426213

[26] Mitra Anita, MacIntyre DA, Paraskevaidi M, Moscicki A-B, Mahajan V, Smith A, Lee YS, Lyons D, Paraskevaidis E, Marchesi JR, Bennett PR, Kyrgiou M. 2021. The vaginal microbiota and innate immunity after local excisional treatment for cervical intraepithelial neoplasia. Genome Med 13:176. doi:10.1186/s13073-021-00977-w 34736529 PMC8567681

[27] Mitra A., MacIntyre DA, Lee YS, Smith A, Marchesi JR, Lehne B, Bhatia R, Lyons D, Paraskevaidis E, Li JV, Holmes E, Nicholson JK, Bennett PR, Kyrgiou M. 2015. Cervical intraepithelial neoplasia disease progression is associated with increased vaginal microbiome diversity. Sci Rep 5:16865. doi:10.1038/srep16865 26574055 PMC4648063

[28] Chen Y, Hong Z, Wang W, Gu L, Gao H, Qiu L, Di W. 2019. Association between the vaginal microbiome and high-risk human papillomavirus infection in pregnant Chinese women. BMC Infect Dis 19:677. doi:10.1186/s12879-019-4279-6 31370796 PMC6669982

[29] Audirac-Chalifour A, Torres-Poveda K, Bahena-Román M, Téllez-Sosa J, Martínez-Barnetche J, Cortina-Ceballos B, López-Estrada G, Delgado-Romero K, Burguete-García AI, Cantú D, García-Carrancá A, Madrid-Marina V. 2016. Cervical microbiome and cytokine profile at various stages of cervical cancer: a pilot study. PLoS ONE 11:e0153274. doi:10.1371/journal.pone.0153274 27115350 PMC4846060

[30] Lee JE, Lee S, Lee H, Song Y-M, Lee K, Han MJ, Sung J, Ko G. 2013. Association of the vaginal microbiota with human papillomavirus infection in a Korean twin cohort. PLoS ONE 8:e63514. doi:10.1371/journal.pone.0063514 23717441 PMC3661536

[31] Guo M, Feng X, Ma J, Zhu K, Niyazi M. 2025. Differential study on the relationship between HPV infection and vaginal microbiota composition in Uygur and Han women. Microb Pathog 198:107149. doi:10.1016/j.micpath.2024.107149 39608511

[32] Brickman CE, Agnello M, Imam N, Camejo P, Pino R, Carroll LN, Chein A, Palefsky JM. 2024. Distinct anal microbiome is correlated with anal cancer precursors in MSM with HIV. AIDS 38:1476–1484. doi:10.1097/QAD.0000000000003920 38691018 PMC11239087

[33] Wu S, Ding X, Kong Y, Acharya S, Wu H, Huang C, Liang Y, Nong X, Chen H. 2021. The feature of cervical microbiota associated with the progression of cervical cancer among reproductive females. Gynecol Oncol 163:348–357. doi:10.1016/j.ygyno.2021.08.016 34503848

[34] Liu H, Liang H, Li D, Wang M, Li Y. 2022. Association of cervical dysbacteriosis, HPV oncogene expression, and cervical lesion progression. Microbiol Spectr 10:e0015122. doi:10.1128/spectrum.00151-22 36036584 PMC9602310

[35] Mancilla V, Jimenez NR, Bishop NS, Flores M, Herbst-Kralovetz MM. 2024. The vaginal microbiota, human papillomavirus infection, and cervical carcinogenesis: a systematic review in the latina population. J Epidemiol Glob Health 14:480–497. doi:10.1007/s44197-024-00201-z 38407720 PMC11176136

[36] Peng Y, Tang Q, Wu S, Zhao C. 2025. Associations of Atopobium, Garderella, Megasphaera, Prevotella, Sneathia, and Streptococcus with human papillomavirus infection, cervical intraepithelial neoplasia, and cancer: a systematic review and meta-analysis. BMC Infect Dis 25:708. doi:10.1186/s12879-025-10851-4 40380083 PMC12082921

[37] Leon-Gomez P, Romero VI. 2024. Human papillomavirus, vaginal microbiota and metagenomics: the interplay between development and progression of cervical cancer. Front Microbiol 15:1515258. doi:10.3389/fmicb.2024.1515258 39911706 PMC11794528

[38] Łaniewski P, Joe TR, Jimenez NR, Eddie TL, Bordeaux SJ, Quiroz V, Peace DJ, Cui H, Roe DJ, Caporaso JG, Lee NR, Herbst-Kralovetz MM. 2024. Viewing native American cervical cancer disparities through the lens of the vaginal microbiome: a pilot study. Cancer Prev Res (Phila) 17:525–538. doi:10.1158/1940-6207.CAPR-24-0286 39172513 PMC11532753

[39] Tsegaye AT, Shing JZ, Vo JB, Kreimer AR, Shiels MS. 2025. Racial and ethnic differences in HPV-related cancer incidence in the United States, 2001-2020. J Natl Cancer Inst:djaf107. doi:10.1093/jnci/djaf107 40289254 PMC12342807

[40] Onywera H, Williamson A-L, Mbulawa ZZA, Coetzee D, Meiring TL. 2019. The cervical microbiota in reproductive-age South African women with and without human papillomavirus infection. Papillomavirus Res 7:154–163. doi:10.1016/j.pvr.2019.04.006 30986570 PMC6475661

[41] Yang C-Y, Chang T-C, Lee Y-T, Shih T-Y, Li C-W, Cheng C-M. 2025. Exploring the interplay between cervicovaginal microbiome, HPV infection, and cervical intraepithelial neoplasia in taiwanese women. J Med Virol 97:e70190. doi:10.1002/jmv.70190 39868896

[42] Zhou Y, Wang L, Pei F, Ji M, Zhang F, Sun Y, Zhao Q, Hong Y, Wang X, Tian J, Wang Y. 2019. Patients with LR-HPV infection have a distinct vaginal microbiota in comparison with healthy controls. Front Cell Infect Microbiol 9:294. doi:10.3389/fcimb.2019.00294 31555603 PMC6722871

[43] Cheng L, Norenhag J, Hu YOO, Brusselaers N, Fransson E, Ährlund-Richter A, Guðnadóttir U, Angelidou P, Zha Y, Hamsten M, Schuppe-Koistinen I, Olovsson M, Engstrand L, Du J. 2020. Vaginal microbiota and human papillomavirus infection among young Swedish women. NPJ Biofilms Microbiomes 6:39. doi:10.1038/s41522-020-00146-8 33046723 PMC7552401

[44] Liu Y, Li T, Guo R, Chen T, Wang S, Wu D, Li J, Liu Z, Zhao Y, Yin J, Qin J, Sun L, Chen W. 2023. The vaginal microbiota among the different status of human papillomavirus infection and bacterial vaginosis. J Med Virol 95:e28595. doi:10.1002/jmv.28595 36811337

[45] Zhang Y, Wu X, Li D, Huang R, Deng X, Li M, Du F, Zhao Y, Shen J, Chen Y, Zhang P, Hu C, Xiao Z, Wen Q. 2024. HPV-associated cervicovaginal microbiome and host metabolome characteristics. BMC Microbiol 24:94. doi:10.1186/s12866-024-03244-1 38519882 PMC10958955

[46] Smith SB, Ravel J. 2017. The vaginal microbiota, host defence and reproductive physiology. J Physiol 595:451–463. doi:10.1113/JP271694 27373840 PMC5233653

[47] Borgdorff H, Tsivtsivadze E, Verhelst R, Marzorati M, Jurriaans S, Ndayisaba GF, Schuren FH, van de Wijgert JHHM. 2014. Lactobacillus-dominated cervicovaginal microbiota associated with reduced HIV/STI prevalence and genital HIV viral load in African women. ISME J 8:1781–1793. doi:10.1038/ismej.2014.26 24599071 PMC4139719

[48] Lewis FMT, Bernstein KT, Aral SO. 2017. Vaginal microbiome and its relationship to behavior, sexual health, and sexually transmitted diseases. Obstet Gynecol 129:643–654. doi:10.1097/AOG.0000000000001932 28277350 PMC6743080

[49] Ozbun MA, Patterson NA. 2014. Using organotypic (raft) epithelial tissue cultures for the biosynthesis and isolation of infectious human papillomaviruses. Curr Protoc Microbiol 34:14B. doi:10.1002/9780471729259.mc14b03s34 PMC4221589 25082004

[50] Herbst‐Kralovetz MM, Quayle AJ, Ficarra M, Greene S, Rose WA II, Chesson R, Spagnuolo RA, Pyles RB. 2008. Quantification and comparison of toll‐like receptor expression and responsiveness in primary and immortalized human female lower genital tract epithelia. American J Rep Immunol 59:212–224. doi:10.1111/j.1600-0897.2007.00566.x 18201283

[51] O’Brien CK, Raskin JR, Amankwa Asare I, Wei C, Ma J, McCoy ZT, Jefferson KK. 2023. Identification of the pore-forming and binding domains of the Sneathia vaginalis cytopathogenic toxin A. PLoS ONE 18:e0284349. doi:10.1371/journal.pone.0284349 37141247 PMC10159106

[52] Fettweis JM, Serrano MG, Sheth NU, Mayer CM, Glascock AL, Brooks JP, Jefferson KK, Consortium VM, Buck GA. 2012. Species-level classification of the vaginal microbiome. BMC Genomics 13:S17. doi:10.1186/1471-2164-13-S8-S17 PMC3535711 23282177

[53] Gao X, Lee V, Prom-Wormley E, Girerd P, Mo H, Xie B, Harwich M, Neale M, York T, Hendricks S, et al.. 2011. The vaginal microbiome: disease, genetics and the environment. Nat Prec. doi:10.1038/npre.2011.5150.2

[54] Wang Q, Garrity GM, Tiedje JM, Cole JR. 2007. Naive bayesian classifier for rapid assignment of rRNA sequences into the new bacterial taxonomy. Appl Environ Microbiol 73:5261–5267. doi:10.1128/AEM.00062-07 17586664 PMC1950982

[55] Gravitt PE, Peyton CL, Alessi TQ, Wheeler CM, Coutlée F, Hildesheim A, Schiffman MH, Scott DR, Apple RJ. 2000. Improved amplification of genital human papillomaviruses. J Clin Microbiol 38:357–361. doi:10.1128/JCM.38.1.357-361.2000 10618116 PMC88724

[56] Parikh HI, Koparde VN, Bradley SP, Buck GA, Sheth NU. 2017. MeFiT: merging and filtering tool for illumina paired-end reads for 16S rRNA amplicon sequencing. BMC Bioinform 17:491. doi:10.1186/s12859-016-1358-1 PMC5134250 27905885

[57] Edgar RC. 2010. Search and clustering orders of magnitude faster than BLAST. Bioinformatics 26:2460–2461. doi:10.1093/bioinformatics/btq461 20709691

[58] Fontan CT, Das D, Bristol ML, James CD, Wang X, Lohner H, Atfi A, Morgan IM. 2020. Human papillomavirus 16 (HPV16) E2 repression of TWIST1 transcription is a potential mediator of HPV16 cancer outcomes. mSphere 5:e00981-20. doi:10.1128/mSphere.00981-20 PMC7729257 33298572

